# The genetic architecture of behavioral traits in a spider

**DOI:** 10.1002/ece3.7430

**Published:** 2021-03-25

**Authors:** Simona Kralj‐Fišer, Jutta M. Schneider, Matjaž Kuntner, Kate Laskowski, Francisco Garcia‐Gonzalez

**Affiliations:** ^1^ Scientific and Research Centre of the Slovenian Academy of Sciences and Arts Institute of Biology Evolutionary Zoology Laboratory Ljubljana Slovenia; ^2^ Institut für Zoologie Fachbereich Biologie Universität Hamburg Hamburg Germany; ^3^ Department of Organisms and Ecosystems Research Evolutionary Zoology Laboratory National Institute of Biology Ljubljana Slovenia; ^4^ University of California Davis One Shields Ave Davis USA; ^5^ Estación Biológica de Doñana‐CSIC Seville Spain; ^6^ Centre for Evolutionary Biology School of Biological Sciences University of Western Australia Western Australia Australia

**Keywords:** animal model, animal personality, heritability, sexual dimorphism, sexual selection

## Abstract

The existence of consistent individual differences in behavior has been shown in a number of species, and several studies have found observable sex differences in these behaviors, yet their evolutionary implications remain unclear. Understanding the evolutionary dynamics of behavioral traits requires knowledge of their genetic architectures and whether this architecture differs between the sexes. We conducted a quantitative genetic study in a sexually size‐dimorphic spider, *Larinioides sclopetarius,* which exhibits sex differences in adult lifestyles. We observed pedigreed spiders for aggression, activity, exploration, and boldness and used animal models to disentangle genetic and environmental influences on these behaviors. We detected trends toward (i) higher additive genetic variances in aggression, activity, and exploration in males than females, and (ii) difference in variances due to common environment/maternal effects, permanent environment and residual variance in aggression and activity with the first two variances being higher in males for both behaviors. We found no sex differences in the amount of genetic and environmental variance in boldness. The mean heritability estimates of aggression, activity, exploration, and boldness range from 0.039 to 0.222 with no sizeable differences between females and males. We note that the credible intervals of the estimates are large, implying a high degree of uncertainty, which disallow a robust conclusion of sex differences in the quantitative genetic estimates. However, the observed estimates suggest that sex differences in the quantitative genetic architecture of the behaviors cannot be ruled out. Notably, the present study suggests that genetic underpinnings of behaviors may differ between sexes and it thus underscores the importance of taking sex differences into account in quantitative genetic studies.

## Graphical abstract

We assessed quantitative genetic estimates of aggression, activity, exploration, and boldness in a sexually‐size‐dimorphic spider. The mean heritability estimates of behaviors range from 0.039 to 0.222 with no sizeable differences between females and males. We detected trends toward higher additive genetic variances in aggression, activity, and exploration in males than in females.

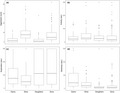

## 1 INTRODUCTION

Individuals commonly differ in behaviors that are consistent over time and/or context, that is, personality traits, coping styles, or temperament (Gosling, 2001; Koolhaas et al., 1999; Réale et al., 2007). Theoretically, consistent variation in behavior has been explained by spatio‐temporal variation in selective pressures often generated by state‐dependent positive feedback loops or negative frequency dependent selection (Wolf et al., 2007; Wolf and Weissing 2012; Sih et al., 2015). To comprehend the past evolution and the potential for continued evolution of these behavioral traits, it is important to study their genetic underpinnings. Most studies show that behavioral traits are moderately heritable, though heritability estimates often differ between traits and taxonomic groups (reviewed in Dochtermann et al., 2019; van Oers & Sinn, 2013). Furthermore, several studies have demonstrated among‐population variation in heritability estimates of individual behavioral traits (Bell, 2005; Dingemanse et al., 2009) suggesting differences in selection pressures among populations. An additional major source of variation in selective pressures can be sex‐specific selection (Schuett et al., 2010). Given that behaviors can act as both targets and mediators of natural and sexual selection processes, it is important to understand that the genetic architectures of behavioral traits and whether this architecture differs between the sexes.

Behavioral traits can affect an individual's survival and reproductive success (Biro & Stamps, 2008; Moiron et al., 2020), and the behavioral strategies to maximize fitness may strongly differ between sexes (Fairbairn et al., 2007; Schuett et al., 2010). Males and females commonly differ in the mean levels of behavior, for example, males are on average more aggressive and bolder than females (Schuett et al., 2010; e.g., Kralj‐Fišer et al., 2017; Kaiser et al., 2018). Sexes may also differ in the degree of behavioral repeatability, that is, a ratio between among‐ and within‐individual variance in a trait, with males, in general, exhibiting higher repeatability in their behavior than females (Bell et al., 2009; Nakagawa et al., 2007). Schuett et al., (2010) suggested that repeatability signals predictability, which might have been selected for through mate choice and male–male competition. Despite emerging links between aspects of individual behavioral variation (mean levels, repeatability) and sexual selection (mate choice, intrasexual competition), the role of sexual selection in the evolution of individual behavioral variation has been rarely explored empirically, though it has recently gathered increased attention (Hämäläinen et al., 2018; Immonen et al., 2018; Tarka et al., 2018). A critical first step to understand the potential role of sex‐specific selection on the evolution of consistent individual behavioral differences is to identify the heritability and underlying genetic architectures (sex‐specific genetic variances, genetic covariance between sexes) of behavioral traits, along with the determination of whether behavioral expression differs between the sexes.

Females and males share the same genes apart from those on heteromorphic sex chromosomes. Thus, the sexes share the genetic basis for most homologous traits. If selection pressures on those traits are opposing in males versus females, shared genetic variance may constrain one or both sexes from reaching their phenotypic optima, setting the stage for intralocus sexual conflict. Theoretically, at least a partial resolution to intralocus sexual conflict is required for the sex‐independent trait expression and evolution of sexual dimorphism. To quantify the amount of sex‐specific genetic variance and genetic divergence of the sexes, the cross‐sex genetic correlation between homologous male and female traits is commonly estimated as $r_{\mathrm{mf}}=\frac{COV_{\mathrm{Amf}}}{\sqrt{V_{\mathrm{Af}}*V_{\mathrm{Am}}}}$, where *COV_Amf_* is the additive genetic covariance between the sexes, and *V_Am_* and *V_Af_* are additive genetic variances of males and females, respectively (Lande, 1980). The cross‐sex genetic correlation measures the extent of similarity between the additive alleles when expressed in both sexes (Bonduriansky & Chenoweth, 2009; Lande, 1980). When *r_mf_* is close to one, a shared trait is assumed to be controlled by a common genetic architecture in both sexes. On the other hand, when *r_mf_* approaches zero, the two sexes differ in the genetic architecture for the shared trait, or differ in allele expression. Sex‐independent evolution in a trait is possible through low additive genetic covariance between the sexes, sex differences in additive genetic variances, or both (Lande, 1980; Lynch & Walsh, 1998). However, when the additive genetic variance of one or both sexes tends to zero (denumerator = 0), the *r_mf_* cannot be estimated.

Despite the potential for between‐sex differences in genetic variation, so far, there are comparatively few studies that have investigated this (but see Han & Dingemanse, 2017; Walling et al., 2008; White et al., 2019). One example comes from southern field crickets, *Gryllus bimaculatus*, where males are more aggressive and more explorative than females. Males also have higher genetic variance for both behaviors, and there is a low cross‐sex genetic correlation for aggression implying a degree of genetic independence for these traits between males and females, and a resolution of sexual conflict to some extent (Han & Dingemanse, 2017). Furthermore, a recent study in an orb‐web spider, *Nuctenea umbratica*, found higher heritability of aggression in males compared with females, though females and males did not differ in additive genetic variation of this trait (Kralj‐Fišer et al., 2019). The two sexes, however, showed no differentiation in genetic architecture of activity (Kralj‐Fišer et al., 2019). Similarly, the quantitative genetic study by White and colleagues (2019) suggested that behavioral traits related to risk‐taking in the Trinidadian guppy, *Poecilia reticulata*, where males are bolder, lack sex‐specific genetic architecture within traits. Namely, the sexes did not differ in the amount of sex‐specific genetic variances and cross‐sex genetic correlations within traits did not differ from unity (White et al., 2019). Nonetheless, there was a weak evidence of sex‐specificity in genetic correlations between traits (White et al., 2019). More research is needed, however, to gain a more general insight into the sex‐specificity of behavioral genetic architectures, sex‐specific selection, and the sexual conflict dynamics of individual behavioral variation.

Here, we examine sex‐specific heritability estimates of a series of behavioral traits, namely aggression, boldness, activity, and exploration in a novel environment in a spider model, *Larinioides sclopetarius*. We used data generated by Kralj‐Fišer and Schneider’s (2012) study, in which the above behaviors were measured in males and females. In that study, the heritability of the different behaviors was assessed using parent–offspring regression, but combined across both sexes (Kralj‐Fišer & Schneider, 2012). Here, we use the animal model (Kruuk & Hadfield, 2007) to test whether females and males differ in quantitative genetic estimates in these traits. We partition the observed phenotypic variance (*V_P_*) in each trait into additive genetic (*V_A_*), common environment/maternal (*V_CE/M_*), permanent environment (*V_PE_*), and residual variances (*V_R_*). In addition, we calculate two mean‐standardized evolvability measures, the coefficient of additive genetic variation and its square, as well as the coefficient of common environment/maternal effect variation, permanent environmental effect variation, and the coefficient of residual variation. These values enable the comparison of components of phenotypic variance and evolvability among different traits, sex, and taxa (Garcia‐Gonzalez et al., 2012; Hansen et al., 2011; Houle, 1992). Another goal is the estimation of the cross‐sex genetic correlation in these traits.

*Larinioides* spiders are sexually size dimorphic and exhibit large sex differences in their adult lifestyles, suggesting sex‐specific selection pressures. Females are territorial, sit‐and‐wait predators, whereas males cease web building after reaching maturity; adult males wander around in search of mates and feed mostly commensally in female webs. Based on these sex differences, we could predict that males exhibit higher mean levels of activity and exploration, but a previous laboratory study on this species failed to support this expectation (Kralj‐Fišer & Schneider, 2012). However, that study also found higher mean repeatability in males compared with females in activity and exploration, but not in boldness (activity; R♂ = 0.894, R♀ = 0.401; exploration, R♂ = 0.734, R♀ = 0.530; boldness, R♂ = 0.798, R♀ = 0.824; Kralj‐Fišer & Schneider, 2012). Given that the repeatability of a trait represents an upper limit for its heritability (Falconer & Mackay, 1996; Lynch & Walsh, 1998), we expect to find that activity and exploration would have higher heritability estimates in males than in females. Furthermore, we predict that selection on activity and exploration might be stronger for males (finding mates) than for females, who would benefit more from saving energy for offspring production, as they do not need to move to feed or find mates. Thus, we would also expect sex‐specificity in the genetic architectures of these traits. We would further expect that males, who move more and may thus be more conspicuous than females, have been under stronger selection for boldness and thus may potentially have different underlying genetic architecture of the trait compared with females.

*Larinioides* males commonly fight for access to mates, while female disputes are rare (Kralj‐Fišer & Schneider, 2012). This was observed also under laboratory conditions, where males were generally more aggressive than females (Kralj‐Fišer & Schneider, 2012). In males, intrasex aggression enhances access to mates and has been likely shaped by sexual selection. In females, aggression toward same‐sex conspecifics serves to defend their territory (web) and foraging patch, and overt aggression may have high fitness costs due to injuries and death (Kralj‐Fišer & Schneider, 2012). The results from previous laboratory experiments imply that aggressive males father more offspring than nonaggressive ones, whereas female aggression is not related to fecundity (Kralj‐Fišer et al., 2013). Based on the repeatability estimates for aggression found in that study (R♂ = 0.864, R♀ = 0.772), we expected that the sexes may not greatly differ in heritability for this trait (Kralj‐Fišer & Schneider, 2012). Nevertheless, we predict to find some sex differences in the underlying genetic architecture, because males and females seem to differ in their phenotypic optima for aggression.

## 2 MATERIAL AND METHODS

### 2.1 Study animals

**FIGURE 1:**
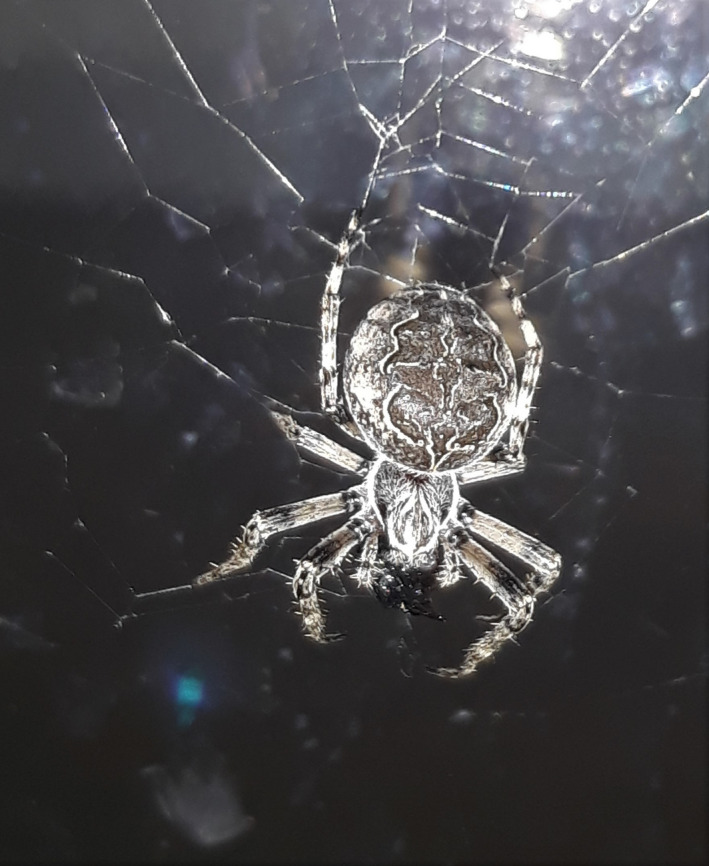
*Larinioides sclopetarius* female. Photograph: Rok Golobinek

*Larinioides sclopetarius* (Figure 1) is a nocturnal, Holarctic orb‐weaving spider, commonly found in high densities on human constructions near water bodies (Heiling & Herberstein, 1998). Individuals build webs adjacent to one another, but retain territorial and aggressive habits. We collected subadult *L. sclopetarius* along riverbanks and bridges in Hamburg (Germany) in September 2010, then transferred them to the laboratory to be reared to adulthood in 200 ml plastic cups and fed with ad libitum *Drosophila sp*. flies. Adult females are larger than males with size dimorphism index of 0.85 (Turk et al. 2019); therefore, we accordingly adjusted the food regime upon maturation; adult females (*N* = 30) that were placed into plastic frames (36 x 36 x 6 cm) were fed with *Calliphora sp*, whereas males (*N* = 31) remained in the 200 ml cups under the same feeding treatment (ad libitum *Drosophila sp*.). We fed the spiders twice a week, misted their webs with water using a spray bottle five days a week, and kept them at room temperature under 10:14 (LD) conditions. Research on spiders is not restricted by the animal protection law in EU. We collected minimal number of individuals in the field to conduct the study. Natural populations of tested spiders are abundant in the field, and their populations are not at risk. Spiders were not harmed in any way.

### 2.2 Experimental design

We used the data from Kralj‐Fišer and Schneider (2012), where additional details may be found. Kralj‐Fišer and Schneider (2012) observed the behavior of adult male and female spiders in a series of standardized personality tests designed to test aggression, activity, exploration, and boldness. Each individual from the parental generation was tested twice in each of the test situations. Aggression was tested by placing two individuals of the same sex approximately 5 cm from each other, and their aggression‐related behaviors, that is, approaching, web shaking, attacking, chasing, and biting, were recorded for 20 min. Opponents were mass‐matched to the average difference of 15.11% and 7.93% in females and males, respectively. Activity and exploration were tested in a novel environment. A focal spider was gently placed in an unfamiliar plastic box (11 x 11 x 6 cm) using a paintbrush and observed for 300 s. When positioned in the box, the spider started walking within the box. The latency to the first pause in walking after being placed into the box was recorded (activity). The pause was considered when the spider stopped moving for at least 2 s. Thereafter, the latency to move again after the first halt (exploration) was recorded. In both cases, 300 s were taken as the maximum latency. Boldness was measured as a response to a simulated predator attack. A spider was placed in the plastic container (11 x 11 x 6 cm) using a soft brush. Then, a predator attack was simulated by shuddering the container until the spider feigned death—a spider posture very similar to that of a dead spider. The time that elapsed between the start of death feigning to the first move afterward was used as boldness. 300 s were taken as the maximum duration.

The animals were then mated assortatively by aggressiveness type. 29 females produced at least one egg‐sac that we collected and stored in a climate chamber at 25°C until hatching. Spiderlings (from the first clutch) were kept together until their second molt (they die if separated before); thereafter, they were separated into individual cups. These offspring were reared under standard conditions as described above. After reaching maturity, their behaviors were measured in the same way as in their parents, but once in each of the tests. The aim was to test 10 full‐siblings: 5 males and 5 females per family (*N* = 29 families), but some clutches were sex‐biased. 262 spiderlings (134 sons, 128 daughters) distributed among the families in the breeding design (mean ± SE of 4.963 ± 0.189 daughters, and 4.741 ± 1.074 sons, per family) were tested.

We note that in some contexts, assortative mating can potentially bias estimates of variance components and heritability. However, if the phenotypes of all individuals including parents are included in an animal model (Walsh & Lynch, 2018), as is the case here, assortative mating, in fact, serves to increase the precision of the estimates (Michael Morrissey unpublished; see Kralj‐Fišer et al., 2019).

### 2.3 Analyses

Selection works simultaneously on multiple (correlated) traits rather on a single trait (Lande & Arnold, 1983). Therefore, exploring sex‐specific genetic (co)variance in a multivariate sense that considers the among‐trait covariance structure is desirable (Walsh & Blows, 2009; Wyman et al., 2013). While the framework for the multivariate approach is available, such studies are rare (e.g., Gosden et al., 2012; White et al., 2019), which may be mainly due to difficulty of recording multiple behaviors in a large number of individuals required in the quantitative genetic studies. In our case, this was precisely a constraint and, therefore, we asses genetic architectures on a trait by trait basis, which still gives an informative and useful, though perhaps restricted, compared to a fully multivariate approach, view on trait evolution.

We used animal models to assess sex‐specific quantitative genetic parameters of the four behaviors: aggression, activity, exploration, and boldness (Wilson et al., 2010). Activity and exploration scores were log transformed prior to the analyses. Animal models are mixed‐effects models that decompose phenotypic variance into genetic and environmental effects (Kruuk & Hadfield, 2007). We ran Markov Chain Monte Carlo Linear Mixed Models using the package MCMCglmm in R (version 3.5.1., R Core Team, 2013, Hadfield, 2010) to partition the observed phenotypic variance (*V_P_*) in a trait into additive genetic (*V_A_*), common environment/maternal (*V_CE/M_*), permanent environment—container ID (*V_PE_*) accounting for the repeated measures of each individual and residual variances (*V_R_*). We used the command *us* to enable estimation of sex‐specific additive genetic variance in a trait (females’ additive genetic variance = *V_Af_*; males’ additive genetic variance = *V_Am_*) and an assessment of the additive genetic covariance in a trait between males and females (*COV_Am_*_f_). We constrained *V_PE_* and *V_R_* cross‐sex covariances to zero using the command *idh*. To define genetic relatedness between individuals, we constructed a pedigree containing each individual included in the experiments. Parents of the P (parental) generation were marked as NA, as P individuals were field‐collected, and their family tree was unknown. Spiderlings from the same clutch could not be separated before the second molt, and thus, siblings shared early common environmental effects. Sibling phenotypes, however, also shared the effects of the same mother (maternal effects). Thus, our study design did not distinguish the components of maternal from common environmental effects.

We estimated the narrow‐sense heritability in each trait as $h^{2}=\frac{V_{A}}{\left(V_{A}+V_{CE/M}+V_{\mathrm{PE}}+V_{R}\right)}$ with 95% credible intervals (CIs) for females and males, separately. In the estimation of the heritability of aggressiveness, we additionally included contest ID (*V*_C_) as a random effect because aggressiveness was scored simultaneously for two individuals in dyadic contests. We then estimated narrow‐sense heritability in aggressiveness as $h^{2}=\frac{V_{A}}{\left(V_{A}+V_{CE/M}+V_{\mathrm{PE}}+V_{R}+V_{C}\right)}$. We planned to calculate cross‐sex genetic correlation, $r_{\mathrm{mf}}=\frac{COV_{\mathrm{Amf}}}{\sqrt{V_{\mathrm{Af}}*V_{\mathrm{Am}}}}$ (Lande, 1980). However, the estimated additive genetic variances of one or both sexes were close to zero for all traits (see results), precluding the ability to estimate with confidence the cross‐sex genetic correlations.

Additionally, we calculated two mean‐standardized evolvability measures, the coefficient of additive genetic variation, $CV_{A}=\frac{\sqrt{V_{A}}}{\text{mean}}$, and its square, $I_{A}=\frac{V_{A}}{mean^{2}}$, the coefficient of common environment/maternal effect variation as $CV_{CE/M}=\frac{\sqrt{V_{CE/M}}}{\text{mean}}$, the coefficient of permanent environmental effect variation as $CV_{\mathrm{PE}}=\frac{\sqrt{V_{\mathrm{PE}}}}{\text{mean}}$ and the coefficient of residual variation as $CV_{R}=\frac{\sqrt{V_{R}}}{\text{mean}}$ (Garcia‐Gonzalez et al., 2012; Houle, 1992). *CV_A_, I_A_, CV_M/CE,_ CV _PE,_* and *CV_R_* and their 95% CI were calculated using the raw data (unstandardized) and phenotypic means, which were always obtained from both the parental and offspring generations. We then calculated the mean differences between the females and males in *CV_A_, I_A_, CV_M/CE,_ CV _PE,_* and *CV_R_* for all posterior estimates and obtained the 95% CI for these differences.

We run models with uninformative priors (see in Supplementary material on https://doi.org/10.5061/dryad.ht76hdrb9). We checked convergence and mixing properties by visual inspection of the chains and checked the autocorrelation values. We ran Heidelberger and Welch's convergence diagnostics to verify that the number of iterations was adequate for chains to achieve convergence. Codes and results of all analyses are given in Supplementary material on https://doi.org/10.5061/dryad.ht76hdrb9.

## 3 RESULTS

### 3.1 Aggression

**FIGURE 2:**
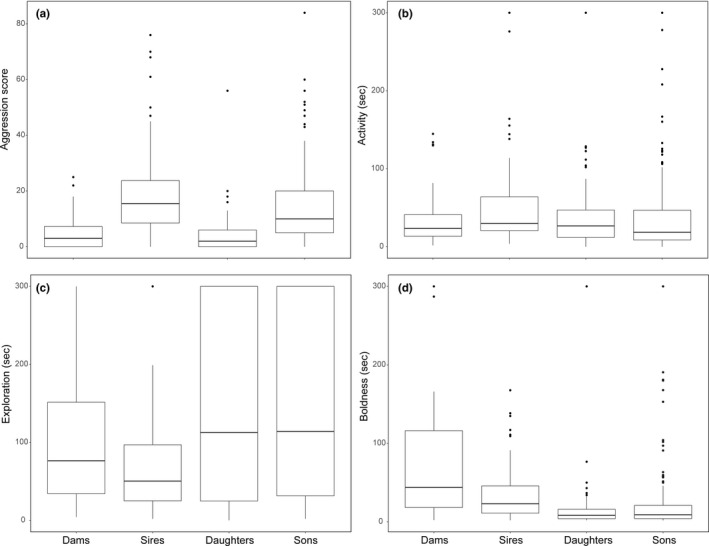
Boxplots show the median and 25th and 75th percentiles of behaviors in dams, sires, daughters, and sons. A) aggression scores, B) activity—initial duration of movement after being introduced into a novel environment, C) exploration—the latency to move again after the first stop, and D) boldness—the time that elapsed between the start of death feigning after simulated predator attack to the first move afterward

**FIGURE 3:**
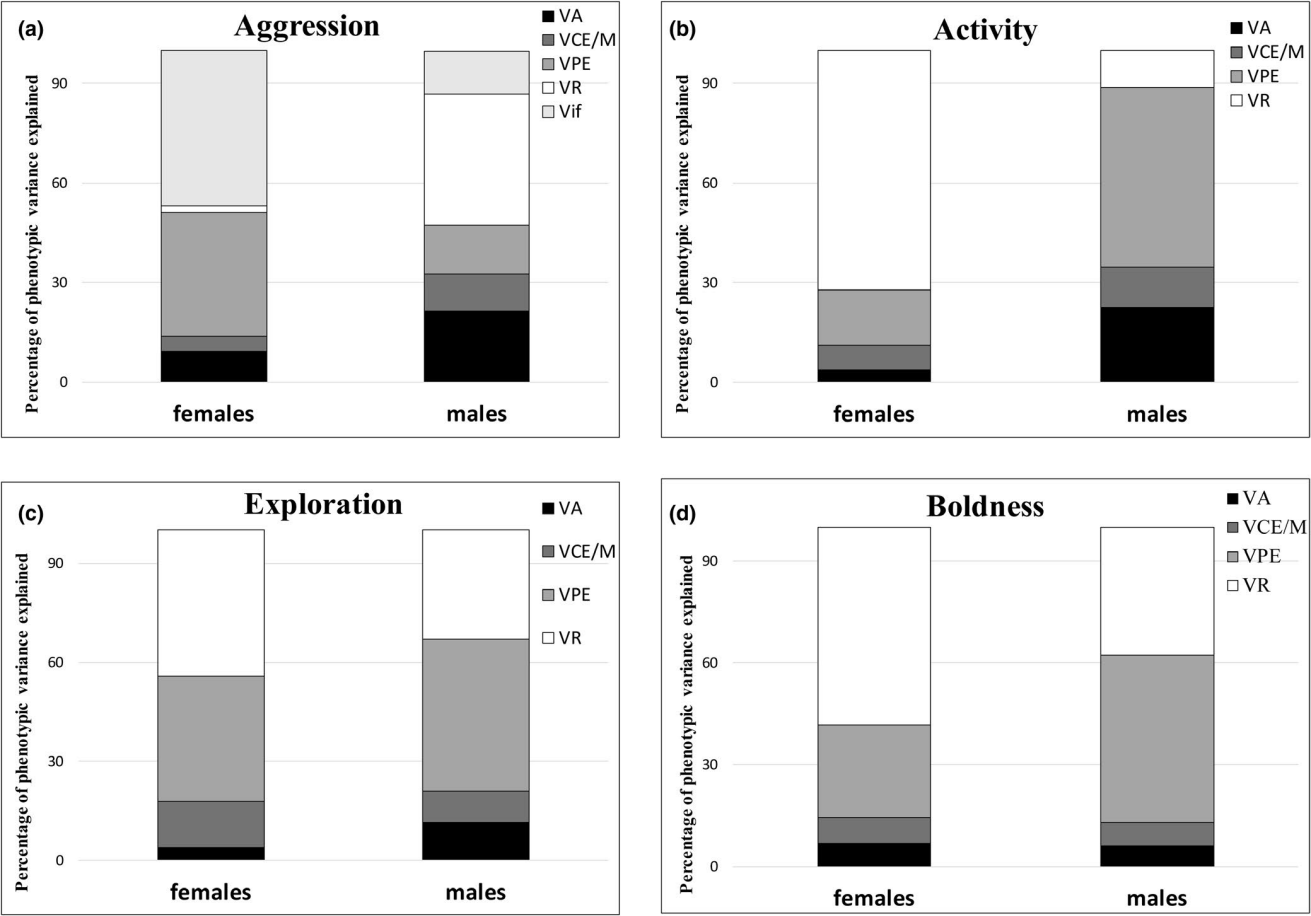
Variance components of behaviors—aggression, activity, exploration, and boldness —in females and males. Stacked bars represent the total phenotypic variance (100%) decomposed into additive genetic variance (*V_A_*), common environment/ maternal effects variance (*V_CE/M_*), permanent environment variance (*V_PE_*), residual variance (*V_R_*), and variance due to contest ID (*V_if_*) in the case of aggression

Females exhibit lower mean aggression compared with males (post. mean difference = −12.104, 95% credible interval (CI) = [−15.773, −8.725]; *p* <.001; Figure 2). The quantitative genetic estimates for females and males are given in Tables 1 and 2. Figure 3a represents the total phenotypic variance (*V_P_*) in aggression scores decomposed into additive genetic variance (*V_A_*), common environment/ maternal effects variance (*V_CE/M_*), permanent environment variance (*V_PE_*), and residual variance (*V_R_*) in females and males, separately. The residual variance of aggression was higher in males than in females (*V_R_*, post. mean difference = −109.659, 95% CI [−168.204, −52.738]). The additive genetic variation (*V_A_*) and common environment/ maternal effects variance (*V_CE/M_*) of aggression tend to be lower in females than in males; differences, however, are not statistically significant, though based on the CIs the existing differences cannot be ruled out, especially in *V_A_* and *V_CE/M_* (*V_A_*, post. mean difference = −56.036, 95% CI [−130.144, 8.505]; *V_CE/M_*, post. mean difference = −29.353, 95% CI [−86.986, 6.446]). We found no sex differences in permanent environment variance (*V_PE_*) (Table 1). The mean heritability of aggression is 0.088, 95% CI [<0.001, 0.261] in females and 0.212, 95% CI [<0.001, 0.440] in males and do not differ significantly between sexes (Table 1). The mean additive genetic covariance between sexes is estimated to posterior mean of 3.109 with 95% CI [−7.236, 15.688].

**TABLE 1:** Estimates (posterior mean (95% credible interval) of the additive genetic variance (*V_A_*), common environment/ maternal effects variance (*V_CE/M_*), permanent environment variance (*V_PE_*), residual variance (*V_R_*), and heritability (*h^2^*). Activity and exploration were log transformed before the analyses. Statistically significant differences between females and males are bolded

Behavior	Sex	VA mean [95% CI]	VCE/M mean [95% CI]	VPE mean [95% CI]	VR mean [95% CI]	h2 mean [95% CI]
Aggression	**♀**	3.330 [<0.001, 10.172]	1.663 [<0.001, 5.726]	13.471 [1.652, 24.723]	0.723 [0.016, 2.190]	0.088 [<0.001, 0.261]
	**♂**	59.367 [<0.001, 130.364]	31.016 [<0.001, 87.849]	41.030 [<0.001, 101.727]	110.382 [54.390, 169.842]	0.212 [<0.001, 0.440]
	**♀ minus ♂**	*−56.036 [−130.144, 8.505]*	*−29.353 [−86.986, 6.446]*	−27.559 [−91.413, 22.930]	**−109.659 [−168.204, −52.738]**	−0.124 [−0.455, 0.171]
Activity	**♀**	0.006 [<0.001, 0.024]	0.012 [<0.001, 0.036]	0.027 [<0.001, 0.068]	0.116 [0.071, 0.160]	0.039[<0.001, 0.144]
	**♂**	0.071 [<0.001, 0.190]	0.038 [<0.001, 0.110]	0.170 [0.074, 0.260]	0.035[0.019, 0.055]	0.222 [<0.001, 0.561]
	**♀ minus ♂**	*−0.064 [−0.195, 0.023]*	*−0.026 [−0.109, 0.035]*	**−0.143 [−0.241, −0.037]**	**0.081 [0.032, 0.130]**	−0.183 [−0.572, 0.125]
Exploration	♀	0.015 [<0.001, 0.058]	0.053 [<0.001, 0.127]	0.144 [<0.001, 0.245]	0.168 [0.078, 0.282]	0.039 [<0.001, 0.148]
	♂	0.051 [<0.001, 0.183]	0.042 [<0.001, 0.120]	0.203 [0.045, 0.341]	0.146 [0.078, 0.231]	0.111 [<0.001, 0.381]
	♀ minus ♂	*−0.036 [−0.195, 0.069]*	0.011 [−0.106, 0.121]	−0.059 [−0.251, 0.132]	0.022 [−0.112, 0.165]	−0.071 [−0.402, 0.161]
Boldness	♀	327.139 [<0.001, 1,162.128]	379.028 [<0.001, 1,401.636]	1,326.757 [<0.001, 2,256.301]	2,852.365 [1930.561, 3,881.258]	0.064 [<0.001, 0.234]
	♂	173.983 [<0.001, 627.098]	208.236 [<0.001, 632.983]	1,431.35 [286.958, 2,422.775]	1,100.852 [484.528, 1970.622]	0.059 [<0.001, 0.209]
	♀ minus ♂	153.155 [−736.497, 1,316.104]	170.792 [−725.994, 1,365.516]	−104.593 [−1681.796, 1,418.35]	1,158.466 [−5572.44, 8,231.994]	0.005 [−0.220, 0.246]

**TABLE 2:** Estimates (posterior mean (95% credible interval) of the coefficient of additive genetic variance (*CV_A_*), common environment/ maternal effects variance (*CV_CE/M_*), permanent environment variance (*CV_PE_*), residual variance (*CV_R_*), and evolvability (*I_A_*). We used the raw behavioral data for the analyses. Statistically significant differences between females and males are bolded

Behavior	Sex	*CV_A_* mean (95% CI)	*CV_CE/M_* mean (95% CI)	*CV_PE_* mean [95% CI]	*CV_R_* mean [95% CI]	*I_A_* mean (95% CI)
Aggression	**♀**	0.352 [<0.001, 0.717]	0.239 [<0.001, 0.538]	0.800 [0.406, 1.179]	0.171 [0.038, 0.336]	0.168 [<0.001, 0.513]
	**♂**	0.442 [0.065, 0.754]	0.301 [<0.001, 0.578]	0.354 [0.001, 0.623]	0.642 [0.468, 0.817]	0.226 [<0.001, 0.496]
	**♀ minus ♂**	−0.090 [−0.609, 0.447]	−0.062 [−0.494, 0.378]	*0.447 [−0.096, 0.960]*	**−0.470 [−0.705, −0.223]**	−0.058 [−0.512, 0.420]
Activity	**♀**	0.161 [<0.001, 0.391]	0.232 [<0.001, 0.471]	0.403 [0.013, 0.697]	0.868 [0.685, 1.041]	0.040[<0.001, 0.153]
	**♂**	0.707 [0.119, 1.251]	0.206 [<0.001, 0.501]	0.976 [0.587, 1.324]	0.592 [0.443, 0.761]	0.575 [<0.001, 1.349]
	**♀ minus ♂**	*−0.546 [−1.143, 0.059]*	0.026 [−0.384, 0.419]	**−0.573 [−1.119, −0.053]**	**0.277 [0.027, 0.471]**	*−0.535 [−1.375, 0.099]*
Exploration	**♀**	0.042 [<0.001, 0.102]	0.061 [<0.001, 0.123]	0.105 [0.001, 0.182]	0.226 [0.178, 0.271]	0.026 [<0.001, 0.099]
	**♂**	0.267 [0.045, 0.473]	0.078 [<0.001, 0.189]	0.369 [0.222, 0.500]	0.224 [0.167, 0.288]	0.077 [<0.001, 0.281]
	**♀ minus ♂**	*−0.225 [−0.433, 0.006]*	−0.017 [−0.156, 0.107]	**−0.264 [−0.441, −0.094]**	*0.003 [−0.078, 0.076]*	−0.051 [−0.298, 0.120]
Boldness	**♀**	0.153 [<0.001, 0.372]	0.221 [<0.001, 0.448]	0.384 [0.013, 0.663]	0.826 [0.652, 0.991]	0.234 [<0.001, 0.904]
	**♂**	1.116 [0.187, 1.974]	0.326 [<0.001, 0.790]	1.540 [0.926, 2.090]	0.934 [0.700, 1.201]	0.174 [<0.001, 0.625]
	**♀ minus ♂**	*−0.963 [−1.815, 0.006]*	−0.104 [−0.666, 0.385]	**−1.157 [−1.877, −0.467]**	−0.108 [−0.423, 0.193]	0.061 [(−0.699, 0.968]

The coefficient of residual variation (C*V_R_*) is lower in females than males, but large CIs preclude firm conclusions (C*V_R_*, post. mean difference = −0.470, 95% CI [−0.705, −0.223]). We found no significant sex difference in C*V_A_* (Table 2). Furthermore, females tend to exhibit higher coefficient of variances due to permanent environmental effect (C*V_PE_* post. mean difference = 0.447, 95% CI [−0.096, 0.969]). We found no sex differences in coefficients of maternal effect (*CV_CE/M_*) (Table 2). We also found no differences between females and males in evolvability (*I_A_*) of aggression (Table 2).

### 3.2 Activity

Males exhibit higher levels of expressed activity in the novel environment than females; however, a sex difference is not significant (*female minus male*: post. mean difference = −13.728, 95% credible interval (CI) = [−29.277, 1.976], *p* =.081; Figure 2). The quantitative genetic estimates, *V_A_*, *V_R_*, *V_CE/M,_* and *V_PE_*, and *h^2^* for females and males are given in Table 1. Figure 3b shows how the *V_P_* in activity is partitioned into in each sex. The results suggest that males tend to have higher *V_A_* in activity than females; the sexes do not differ in this estimate, but the confidence limits indicate that a difference cannot be ruled out with confidence (post. mean difference = −0.064, 95% CI [−0.195, 0.023]). Furthermore, males exhibit higher values of *V_CE/M_* and *V_PE_* compared with females (*V_CE/M_,* post. mean difference = −0.026, 95% CI [−0.109, 0.035]; *V_PE_*, post. mean difference = −0.143, 95% CI [−0.241, −0.037]), whereas females have higher *V_R_* (post. mean difference = 0.081, 95% CI [0.032, 0.130]). The mean heritability of activity is 0.039, 95% CI [<0.001, 0.144] in females and 0.222, 95% CI [<0.001, 0.561] in males and do not differ significantly between sexes (Table 1). The posterior mean additive genetic covariance across sexes (*COV_Am_*_f_) is estimated to < −0.001 with 95% CI between −0.018 and 0.020.

We found a trend toward a higher values of *CV_A_* and *I_A_* in males compared with females (*CV_A_,* post. mean difference = −0.546, 95% CI [−1.143, 0.006]; *I_A_*, post. mean difference = −0.535, 95% CI [−1.375, 0.099]). Furthermore, males have higher *CV_PE_* than females (post. mean difference = −0.573, 95% CI [−1.119, −0.053]), whereas females have higher *CV_R_*. (post. mean difference = 0.277, 95% CI [0.027, 0.471]). Estimates of *CV_CE/M_* do not differ between the sexes (Table 2). While Heidelberger and Welch's diagnostic for the estimation of the additive genetic covariance across sexes passed Stationary test, it did not pass Halfwidth Mean test even with increased iterations. The latter results should be, therefore, taken with caution.

### 3.3 Exploration

There are no sex differences in the expressed level of exploratory behavior (post. mean difference = 7.113, 95% CI [−23.744, 39.101, *p* =.66]; Figure 2). We found a trend toward higher *V_A_* in males compared with females (post. mean difference = −0.036, 95% CI [−0.195, 0.069). None of the other quantitative genetic estimates, *V_R_*, *V_CE/M,_* and *V_PE_* differ significantly between the sexes (Table 1; Figure 3c). Heritability estimates are 0.039, 95% CI [<0.001, 0.148] and 0.111, 95% CI [<0.001, 0.381] for females and males, respectively, and there are no sex differences in the *h^2^* of this trait (Table 1). The estimate of the posterior mean additive genetic covariance across sexes is −0.002 with 95% CI between −0.028 and 0.020.

Furthermore, *CV_A_* is lower in females; the difference is not statistically significant, but based on the CI this difference cannot be ruled out (post. mean difference = −0.225, 95% CI [−0.433, 0.006]. We found that males exhibit significantly higher *CV_PE_* compared with females (post. mean difference = ‐ 0.264, 95% CI [−0.441, −0.094]). We found no sex difference in *CV_CE/M_*, *CV_R_* or *I_A_* (Table 2). Heidelberger and Welch's diagnostic for the estimation of the additive genetic covariance across sexes passed Stationary test, whereas it did not pass Halfwidth Mean test even with increased iterations. The latter results should be, therefore, taken with caution.

### 3.4 Boldness

We found no sex differences in boldness levels (post. mean difference = 5.752, 95% CI [−11.237, 23.417, *p* =.516]; Figure 2). None of the quantitative genetic estimates, *V_A_*, *V_R_*, *V_CE/M,_* and *V_PE_* differ significantly between the sexes (Table 1; Figure 3d). Heritability estimates are 0.064, 95% CI [<0.001, 0.148] and 0.059, 95% CI [<0.001, 0.209] for females and males, respectively. The posterior mean additive genetic covariance across sexes is 23.220, 95% CI [−168.884, 267.603].

We found, however, a trend toward higher *CV_A_* and *CV_PE_* in males compared with females (*CV_A_*, post. mean difference = −0.963, 95% CI [−1.815, 0.006]; *CV_PE_*, post. mean difference = −1.157, 95% CI [−1.877, −0.467]). The sex difference in *CV_A_* is not significant, but based on CIs this difference cannot be ruled out. The estimates of sex difference in *CV_CE/M_*, *CV_R,_* and *I_A_* are not significant (Table 2).

## 4 DISCUSSION

Our study in a sexually size dimorphic spider, *Larinioides sclopetarius,* explored sex‐specific quantitative genetic estimates for several important behaviors. We reported (i) heritability estimates of personality traits—aggression, activity, exploration, and boldness ranging from 0.039 to 0.222 with no sizeable differences between females and males; (ii) a trend toward higher additive genetic variances in aggression, activity, and exploration in males than in females; and iii) a trend toward a difference in values of variances due to common environment/maternal effects, permanent environment, and residual variance in aggression and activity with the first two variances being higher in males for both behaviors; iv) we detected no sex differences in the amount of genetic and environmental variances in boldness. Our plan to estimate the cross‐sex genetic correlations of behaviors failed because the estimated additive genetic variances of one or both sexes were close to zero for all traits, precluding a meaningful estimation. We note that the credible intervals of the estimates are large, implying a high degree of uncertainty, which impedes making robust conclusions about sex differences in the quantitative genetic estimates. On the other hand, the observed estimates suggest that sex differences in the quantitative genetic architecture of the behaviors cannot be ruled out.

We predicted to find a sex‐specific genetic architecture underlying aggressive behavior. Our results suggest that higher additive genetic variance in males compared with females in this trait is likely. Higher additive genetic variance of aggression implies that this trait could have a higher potential to respond to selection in males compared with females. Males also tended toward a higher variance due to common environment/maternal effects and a higher residual variance than females. Thus, males tend to show higher variance in both genetic and environmental variances of aggression than females. This pattern, namely high additive genetic and nongenetic variability, has been suggested for traits closely related to fitness including life‐history traits and sexually selected traits (Houle, 1992; Price and Schluter 1991; Rowe and Houle 1996; Merila & Sheldon, 1999). In *L. sclopetarius*, male aggression and fitness components are related (Kralj‐Fišer et al., 2013). Specifically, under laboratory conditions, aggressive males sire more offspring than nonaggressive ones, while aggression is not related to fecundity in females (Kralj‐Fišer et al., 2013). Based on this evidence, intrasex aggression might affect fitness components to a higher extent in males than in females. Thus, although it is expected that strong directional selection (including sexual selection) should erode genetic variance, genic capture could explain the maintenance of genetic variance in aggressiveness if this trait is linked to fitness and is affected by many loci (Houle, 1992; Price and Schluter 1991; Rowe and Houle 1996; Merila and Sheldon 1999).

Furthermore, high levels of genetic variance in sexually selected traits would be expected whether these traits represent larger mutation targets. Rowe and Houle (1996) suggested that high genetic variation in sexually selected traits, which are often costly to express and condition dependent, reflects the underlying genetic variance in condition. In *L. sclopetarius*, male–male combats that ultimately determine access to mates are common, meaning that it is possible that sexual selection might have shaped intrasex aggression in males. In females, aggression levels toward same‐sex conspecifics, which is used to defend the territory and the foraging patch, are much lower. In both sexes, aggression is subjected to trade‐offs; overt aggression is costly due to injuries and deaths (Kralj‐Fišer & Schneider, 2012). The spiders for this study were weight‐matched when tested for aggression, which prevents analysis of the relationship between body condition and aggression. In most spiders, however, larger males readily win the fights (Hoefler, 2007). In short, the role of selection and genic capture underlying the observed genetic architecture of aggression in our system cannot be defined with certainty. Our data on differences in the residual variance of aggression between the sexes are not conclusive enough to resolve this issue. Had we observed clear larger CV_R_ in aggression for males, this would have added support to a role for genic capture underlying aggressiveness, either through a potential link with body condition and sexual selection, or through a link with fitness, in general. This was, however, not the case because small sample sizes and large CIs hindered our ability to infer processes at play. We need to be cautious, therefore, and additional studies in the laboratory and in wild populations are needed to draw conclusions with confidence. Such studies should also test if aggression in *L. sclopetarius* males is condition‐dependent and whether it is or has been subjected to sexual selection.

We predicted that males, which exhibit higher repeatability in activity and exploration (Kralj‐Fišer & Schneider, 2012), should have higher heritability in these two traits (Falconer & Mackay, 1996). As predicted, males tend to have higher additive genetic variances in both, activity and exploration compared with females (Table 1). Sex differences in genetic variances in activity and exploration suggest that selection pressures acting on this trait might differ between the sexes and implies a higher potential for a response in this trait to selection in males than in females. The result is not surprising given the life‐style differences between adult orb‐web females and males. Females are rather passive sit‐and‐wait predators, whereas males wander around actively searching for mates but cease web building and foraging. It would be interesting to extend this kind of tests to the juvenile stages, when both sexes behave as sit‐and‐wait predators.

We found no sizeable differences in the estimates of sex‐specific genetic and environmental variances of boldness (Table 1). These results imply a similar potential for evolutionary responses to selection in boldness in both sexes. Biologically, we might expect that males and females have a comparable propensity for risk‐taking behaviors (boldness) whether they equally increase their access to resources or chances of survival. Along the same lines, selection on boldness may not differ between the sexes.

Across species, most of the few previous studies investigating sex‐specific genetic variances in aggression have found higher amounts of genetic variance for male compared with female aggression (Table 3). Again, this suggests that sexual selection, specifically male—male competition, likely shapes the genetic architecture of aggression in a sex‐specific way in a variety of species. While the results on sex‐specific genetic variances of activity and exploration are mixed, most studies found no sex differences in the amount of genetic variance of boldness (Table 3). Notably, if a sex difference in a behavior was detected, it consistently indicated higher additive genetic variances in males than in females (Table 3). This is in agreement with the findings of Wyman and Rowe (2014), who showed male‐biased coefficients of additive genetic variance when analyzing sex differences in the amount of genetic variation across a wide range of traits and species. Additional studies are needed to pinpoint general patterns regarding sex‐specific genetic effects in behavioral traits.

**TABLE 3:** Sex differences in additive genetic variance (*V_A_*), and heritability (*h^2^*) for behaviors related to personality

		*V_A_*	*h^2^*	Reference
Aggression	*Gryllus bimaculatus*	♂ > ♀	♂ > ♀	Han & Dingemanse, 2017
	*Nuctenea umbratica*	no sex diff.	♂ > ♀	Kralj‐Fišer et al., 2019
	*Larionioides sclopetarius*	trend for ♂ > ♀	no sex diff	this study
Activity	*Nuctenea umbratica*	no sex diff.	no sex diff.	Kralj‐Fišer et al., 2019
	*Larionioides sclopetarius*	trend for **♂ > ♀**	**♂ > ♀**	this study
Exploration	*Parus major*	no sex diff.	no sex diff.	van Oers et al., 2005
	*Gryllus bimaculatus*	**♂ > ♀**	**♂ > ♀**	Han & Dingemanse, 2017
	*Larionioides sclopetarius*	trend for **♂ > ♀**	no sex diff.	this study
Boldness/risk‐taking	*Parus major*	no sex diff.	no sex diff.	van Oers et al., 2005
	*Poecilia reticulata*	no sex diff.	no sex diff.	White et al., 2019
	*Larionioides sclopetarius*	no sex diff.	no sex diff.	this study

A proximate explanation for the patterns observed may be based on sex differences in chromosome number (karyotype). Most orb‐web spider species exhibit the so‐called X_1_X_2_0 system with X_1_X_2_ males and X_1_X_1_X_2_X_2_ females (Kořínková & Král, 2013). In *L. sclopetarius,* however, sex is determined by an X0 sex‐chromosome system where males are heterogametic with one copy of X chromosome (X0) and females are homogametic with two copies of X chromosome (XX) (Araujo et al., 2012). According to the sex‐chromosome hypothesis, individuals of the heterogametic sex should be more variable in phenotype and genes than individuals of the homogametic sex (Reinhold & Enqvist, 2013). Thus, if genes related to expression of aggression, activity and exploration are sex‐linked, male heterogamety might explain higher additive genetic variance in males (Reinhold & Enqvist, 2013). It should be noted, however, that sex‐chromosome system could not explain sex differences in additive genetic, nonadditive genetic, or phenotypic variances in a recent meta‐analysis (Wyman and Rowe, 2014).

Permanent environmental effects influenced the expression of aggression, activity, exploration, and boldness to a high degree; that is, they explained 15% – 45% of the total phenotypic variances (Table 1, Figure 3). Comparably, a quantitative genetic study found high permanent environmental effects (65% – 85%) of exploration in sticklebacks (Dingemanse et al., 2012), and 18% and 5% of variance in exploration and aggression, respectively, in crickets (Han & Dingemanse, 2017). High repeatability estimates in behaviors of *Larinioides* (Kralj‐Fišer & Schneider, 2012) may be explained largely by permanent environmental effects, that is, paternal effects, epigenetics, nonadditive genetic effects (e.g., dominance variance (Wilson et al., 2010)), and environmental effects that have long‐term effects on phenotypes (nutrition during development, etc.). Hence, consistent individual differences in behaviors may also be due to state‐dependent positive feedback. Notably, activity and exploration covary with spider weight in the offspring generation (Kralj‐Fišer & Schneider, 2012) suggesting that a part of individual variability in these behaviors may be explained by differences in weight.

Maternal effects, that is, the mother's influence on offspring phenotype beyond direct gene transmission, may importantly affect phenotypic variance within a population. A recent meta‐analysis by Moore et al., 2019 reports that maternal effects account for about 10% phenotypic variation within populations, which is half as much as do additive genetic effects. These effects are similar between invertebrate and vertebrate species (Moore et al., 2019). In our study, hatchlings from the same clutch were reared in the same environment until the second molt disallowing to separate maternal from common environmental effects. Thus, we accounted for the resemblance among siblings from the same mother stemming from the same early environment by including mother identity/common environment as a random effect in the models. This explained ~ 5% – 15% of the variance in aggression, activity, exploration, or boldness. Additional research on maternal effects underlying behavioral traits, and specifically personality traits, is necessary to generally assess the importance of these indirect genetic influences underlying individual behavioral variation.

Our heritability estimates of behaviors using the animal model approach differ considerably from the published results from mid‐parent mid‐offspring regression (Kralj‐Fišer & Schneider, 2012). While the previous analyses revealed “significant” heritability only for aggression, and a tendency for heritability in boldness (Kralj‐Fišer & Schneider, 2012), the animal model used here found that the investigated traits have heritability estimates ranging from nearly zero to 0.222 (Table 1). The discrepancy in the results obtained through mid‐parent mid‐offspring regression versus animal model is not unusual (De Villemereuil et al., 2013; Kruuk, 2004). Comparing studies that used both methods, Kruuk (2004) found heritability and standard errors estimated from animal model analyses to be lower than those from the parent–offspring regression or full‐sib analyses. Kruuk (2004) attributed these differences to other sources of variance not accounted for in the simpler techniques. However, Åkesson and colleagues 2008 criticized that conclusion, as Kruuk (2004) evaluated not only two approaches, but also different datasets. A subsequent comparison of the animal model and parent–offspring regression methods on the same data set found no general patterns (De Villemereuil et al., 2013). The differences between the two approaches are, therefore, difficult to assess (De Villemereuil et al., 2013). Our result on behavioral heritability using the same data set with different statistical approaches (Kralj‐Fišer & Schneider, 2012 versus. this study) implies that the animal model may be a more precise approach to reveal the genetic underlying of the measured traits than parent–offspring regression. The latter does not partition the components of variance in a trait, such as maternal, common environmental, and permanent environmental effects, which is what likely renders the behavioral heritability higher (Kralj‐Fišer & Schneider, 2012). In any case, the present study clearly shows that genetic underpinnings of behaviors may differ between sexes, and thus, it underscores the importance of taking sex differences into account in quantitative genetic studies.

## CONFLICT OF INTEREST

The authors declare that they have no conflict of interest.

## AUTHOR CONTRIBUTION

**Simona Kralj‐Fišer:** Conceptualization (lead); Data curation (lead); Formal analysis (lead); Funding acquisition (lead); Investigation (lead); Methodology (equal); Project administration (lead); Writing‐original draft (lead); Writing‐review & editing (lead). **Jutta Schneider:** Funding acquisition (equal); Writing‐review & editing (equal). **Matjaz Kuntner:** Funding acquisition (equal); Writing‐review & editing (equal). **Laskowski Kate:** Writing‐review & editing (equal). **Francisco Garcia‐Gonzalez:** Methodology (equal); Supervision (equal); Writing‐review & editing (equal).
